# Predicting the risk of endometrial cancer in postmenopausal women presenting with vaginal bleeding: the Norwich DEFAB risk assessment tool

**DOI:** 10.1038/sj.bjc.6605620

**Published:** 2010-03-30

**Authors:** N Burbos, P Musonda, I Giarenis, A M Shiner, P Giamougiannis, E P Morris, J J Nieto

**Affiliations:** 1 Department of Obstetrics and Gynaecology, Norfolk and Norwich University Hospital NHS Foundation Trust, Colney Lane, Norwich NR4 7UY, UK; 2 School of Medicine, Health Policy & Practice, University of East Anglia, Norwich NR4 7TJ, UK; 3 Lawson Road Surgery, Lawson Road, Norwich NR3 4LE, UK

**Keywords:** endometrial, risk, postmenopausal, prediction, model

## Abstract

**Background:**

This study aimed to show the longitudinal use of routinely collected clinical data from history and ultrasound evaluation of the endometrium in developing an algorithm to predict the risk of endometrial carcinoma for postmenopausal women presenting with vaginal bleeding.

**Methods:**

This prospective study collected data from 3047 women presenting with postmenopausal bleeding. Data regarding the presence of risk factors for endometrial cancer was collected and univariate and multivariate analyses were performed.

**Results:**

Age distribution ranged from 35 to 97 years with a median of 59 years. A total of 149 women (5% of total) were diagnosed with endometrial carcinoma. Women in the endometrial cancer group were significantly more likely to be older, have higher BMI, recurrent episodes of bleeding, diabetes, hypertension, or a previous history of breast cancer. An investigator best model selection approach was used to select the best predictors of cancer, and using logistic regression analysis we created a model, ‘Norwich DEFAB’, which is a clinical prediction rule for endometrial cancer. The calculated Norwich DEFAB score can vary from a value of 0 to 9. A Norwich DEFAB value equal to or greater than 3 has a positive predictive value (PPV) of 7.78% and negative predictive value (NPV) of 98.2%, whereas a score equal to or greater than 5 has a PPV of 11.9% and NPV of 97.8%.

**Conclusion:**

The combination of clinical information with our investigation tool for women with postmenopausal vaginal bleeding allows the clinician to calculate a predicted risk of endometrial malignancy and prioritise subsequent clinical investigations.

Postmenopausal bleeding refers to any genital tract bleeding in a postmenopausal woman, other than the expected bleeding that occurs in women taking sequential hormone replacement therapy (HRT). Because postmenopausal bleeding is the most common symptom of endometrial cancer, when postmenopausal bleeding occurs, clinical evaluation is indicated (Goldstein *et al*, 2001). Approximately 10% (range 1–25%) of women presenting with postmenopausal bleeding will be diagnosed with endometrial carcinoma (Gambrell *et al*, 1978; Alberico *et al*, 1989; Iatrakis *et al*, 1997). Endometrial atrophy is the most common cause of genital tract bleeding among postmenopausal women (Iatrakis *et al*, 1997). Endometrial hyperplasia and polyps are also common causes.

Two different forms of endometrial carcinoma have been identified. Type-I cancers have an endometrioid histology and account for 70–80% of endometrial carcinomas. They are associated with unopposed oestrogen stimulation of the endometrium and tend to arise in women with obesity, hyperlipidaemia, and other hyper-oestrogenic conditions. Type-II cancers have a non-endometrioid histology and arise in women who are less likely to have the clinical associations seen in type-I cancers (Bokhman, 1983). Several risk factors such as obesity, tamoxifen use, increasing age, hypertension, diabetes, and unopposed use of exogenous oestrogens are strongly associated with increased risk of type-I endometrial cancer (Persson *et al*, 1989; Soler *et al*, 1999; Cohen, 2004; Lachance *et al*, 2006; Friberg *et al*, 2007; Lucenteforte *et al*, 2007; Renehan *et al*, 2008). Early menarche and late menopause have also been implicated due to prolonged oestrogen stimulation of the endometrium. Nulliparity as an isolated risk factor does not appear to increase the risk of endometrial cancer, although due to the high frequency of anovulatory cycles there may be an association in women with subfertility (Chen and Berek, 2008). Hereditary non-polyposis colorectal cancer is a significant but rare risk factor, with descendants of an affected family member carrying a theoretical 50% lifetime risk of endometrial cancer (Aarnio *et al*, 1995).

Currently, controversy exists as to whether transvaginal ultrasonography or endometrial biopsy should be used as the initial diagnostic step for clinical evaluation of women presenting with postmenopausal bleeding (Goldstein *et al*, 2001). In addition, decisions made about the most appropriate investigations that need to be performed, are not always guided by clinical history. The few studies that attempt to include information gained from clinical history to predict the risk of endometrial carcinoma are too small to develop a predictive model (Weber *et al*, 1999; Bachmann *et al*, 2003).

The aim of our study was to use routinely collected clinical data from history and ultrasound evaluation of the endometrium to develop an algorithm to predict the risk of endometrial carcinoma in women presenting to secondary care with postmenopausal vaginal bleeding.

## Materials and methods

### Participants

This is a prospective cohort study, conducted at a gynaecological oncology centre in the United Kingdom, between February 2006 and May 2009. All postmenopausal women presenting with vaginal bleeding to the postmenopausal bleeding clinic were included. Menopause was defined as at least 12 months of spontaneous amenorrhoea. Premenopausal women were not included in the study as there is no standard threshold for endometrial thickness in this group that is considered abnormal. Other groups of women seen at the clinic that were excluded from the study included asymptomatic women with an incidental finding of increased endometrial thickness on imaging and asymptomatic women with abnormal endometrial cytology found on cervical smear.

### Procedures

All women presenting with vaginal bleeding underwent transvaginal ultrasound scanning to evaluate the endometrium. The double-wall endometrial thickness was measured in an anterioposterior dimension from one basalis layer to the other. In keeping with departmental guidelines, when endometrial thickness was measured to be less than 5 mm no further investigations were performed as evidence suggests a low probability of cancer below this threshold (Karlsson *et al*, 1995; Smith-Bindman *et al*, 1998). For the purpose of the study, we considered all women with endometrial thickness less than 5 mm as negative for endometrial cancer.

Women with endometrial thickness equal to or greater than 5 mm had endometrial sampling performed using an endometrial Pipelle device. Hysteroscopic evaluation of the endometrium with biopsy under a general anaesthetic was performed if Pipelle biopsy was not possible or did not yield sufficient tissue for histological diagnosis. A hysteroscopy was also performed for any woman re-appearing at the clinic for a second time with a recurrent episode of bleeding.

### Clinical risk factors – data collection

The clinic collects routine data regarding essential clinical information and presence of risk factors for endometrial cancer using a pre-designed proforma. Data extracted from these forms for this study were age of the patient at presentation, body mass index (BMI), use of HRT, presence of hypertension and diabetes, previous history of breast cancer, and use of tamoxifen. Endometrial thickness measured on ultrasound scan and results of histology when performed were also recorded. We excluded data regarding parity as we consider that it is the frequency of anovulatory cycles that increases the risk of endometrial cancer and not nulliparity *per se*. Data from 90% of the patients were collected prospectively and only in 10% of the cases was it collected retrospectively.

We also attempted to assess whether the bleeding pattern of women had any predictive value in the histological outcome. The amount of bleeding was characterised as spotting, light (=less than a period), and heavy (=like a period or worse). Any event lasting less than 7 days was defined as a single bleeding episode. Recurrent episodes were defined as any bleeding episode lasting 7 or more days or two or more separate bleeding events within the last 12 months.

All the data analysed were collected as part of the routine investigations and treatment. The patients were investigated according to established evidence-based departmental guidelines.

### Statistical analysis

The distributions of continuous variables were not symmetric. To test for normality, the Shapiro-Wilk W-test was used, as was the q-q plot to investigate normality graphically (results not shown). There was no evidence to suggest that data were normally distributed, hence in the descriptive statistics for continuous variables, we report median and inter-quartile range. To avoid inflating the type-I error rate, loss of power, residual confounding, and bias, continuous predictor variables were not categorised (Del Priore *et al*, 1997; Austin and Brunner, 2004; Royston *et al*, 2006). To test any differences we used a non-parametric Wilcoxon rank sum (Mann–Whitney) test. Binomial exact methods were used to calculate 95% confidence intervals (CIs) of the proportions and to test any differences in the proportions observed. *χ*^2^-test was used after checking the expected assumptions. An investigator best model selection approach was used to select the best predictors of cancer in the multiple logistic regression model as opposed to machine-led step-wise regression, which is not advisable (Hurvich and Tsai, 1990; Derksen and Keselman, 1992). Selection of predictor variables was performed by using the likelihood ratio test after estimation of the nested models by adding and eliminating variables one at a time. The likelihood ratio test is similar to using model selection indices such as Akaike information criterion (AIC) or Bayesian information criterion (BIC). All analyses were performed using STATA software, version 10.1 SE (Stata Corporation, College Station, TX, USA).

## Results

### Demographics

During a 39-month interval, 3047 women were investigated for postmenopausal vaginal bleeding. Age distribution ranged from 35 to 97 years with a median of 59 years. A total of 149 women (5% of total) were diagnosed with endometrial carcinoma. Women with all types of endometrial cancer were included in this group. The remaining 2898 women (95%) were included in the non-cancer group for the purposes of the study.

### Clinical risk factors

The results of univariate analysis to assess for correlation between individual clinical characteristics and development of endometrial cancer are given in Table 1. Women in the endometrial cancer group were significantly older (median 64 *vs* 59 years; *P*<0.0001) and had higher BMI (31 *vs* 28 kg m^−2^, *P*<0.0001) than women without cancer. They were more likely to have diabetes (*P*<0.0001) and hypertension (*P*=0.001). The duration of use of HRT did not appear to increase the risk of endometrial cancer (*P*=0.243). The women in the endometrial cancer group were significantly more likely to have a previous history of breast cancer (*P*=0.025). However, the duration of use of tamoxifen in the breast cancer group did not appear to increase the risk of endometrial cancer (*P*=0.091). The amount of vaginal bleeding did not appear to be associated with increased risk of endometrial cancer (*P*=0.289). Recurrent episodes of vaginal bleeding were significantly more likely to be associated with endometrial cancer than a single bleeding event (*P*<0.0001). Endometrial thickness on ultrasound scan was significantly higher in women with endometrial cancer (14.9 *vs* 4.6 mm; *P*<0.0001).

As a result of the statistical analysis the investigating team determined that the factors considered best predictors of endometrial malignancy were age, BMI, presence of diabetes, and endometrial thickness (*P*-value <0.0001, 0.038, 0.030, and <0.0001, respectively). Recurrent episodes of vaginal bleeding were significantly more likely to be associated with endometrial cancer than a single episode (odds ratio 3.93, 95% CI 2.48–6.23), taking into account diabetic status, age, BMI, and endometrial thickness (Table 2).

### Predictive model: Norwich DEFAB

We have created a model with regard to predicting the odds of endometrial carcinoma in postmenopausal women presenting with vaginal bleeding. We are calling this tool DEFAB, which is a clinical prediction rule based on Diabetes, Endometrial thickness, Frequency of bleeding, Age, and BMI. In the DEFAB criteria, presence of diabetes in a patient scores 2; endometrial thickness ⩾14 mm scores 1; recurrent episodes of bleeding scores 4; age ⩾64 years scores 1; and BMI⩾31 kg m^−2^ scores 1. If a criterion is absent, then the score is 0. The calculated Norwich DEFAB score can vary from a value of 0–9. The scores were arrived at by taking account of the predictive odds of cancer from the adjusted model.

The overall sensitivity, specificity, and likelihood ratio for each Norwich DEFAB cut-off point are shown in Table 3. Table 3 also shows the overall proportion (percentage) of the total numbers that have been correctly classified by Norwich DEFAB in each category. The difference in the odds for malignancy predicted by a Norwich DEFAB value equal to or greater than 3 and equal to or greater than 5 was 4.53 and 6.06, respectively. Table 4 shows the sensitivity, specificity, positive predictive value (PPV), negative predictive value (NPV), and receiver operating characteristics (ROC) area for Norwich DEFAB cut-off values equal to or greater than 3 and 5. A Norwich DEFAB value equal to or greater than 3 achieved a sensitivity of 81.9% (95% CI, 74.7–87.7%), specificity of 50.1% (95% CI, 48.2–51.9%), and an ROC area of 0.660 (95% CI, 0.627–0.692). For a Norwich DEFAB cut-off score equal to or greater than 5, sensitivity, specificity, and ROC area were 67.8% (95% CI, 59.6–75.2%), 74.1% (95% CI, 72.5–75.7%), and 0.710 (95% CI, 0.671–0.748), respectively.

**Figure 1:**
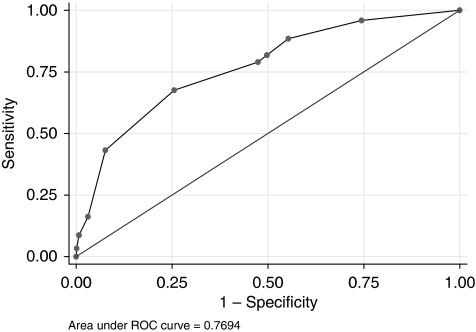
Area under the ROC curve for DEFAB scores.

The accuracy of a test depends on how well the test separates the group being tested into those with and without the disease in question. The area under the ROC curve measures accuracy. An area of 1 represents a perfect test and an area of 0.5 represents a worthless test. The overall predictive ability for the Norwich DEFAB measured by the area under the ROC curve was 0.7694 (Figure 1). Our clinical prediction rule would be considered to be of ‘fair accuracy’ at separating women with cancer from women without cancer, according to the traditional academic point system: fail, poor, fair, good, excellent.

## Discussion

The main objective of the diagnostic evaluation of women with postmenopausal vaginal bleeding is exclusion of malignancy. Women with postmenopausal uterine bleeding may be assessed initially with either endometrial biopsy or transvaginal ultrasonography. Initial evaluation does not require performance of both tests (ACOG Committee Opinion No 440, 2009). Currently, with respect to mortality, morbidity, and quality-of-life end points, there are insufficient data to comment as to whether transvaginal ultrasonography or endometrial biopsy is most effective for initial evaluation of this group of women. Which approach is used initially depends on the risk of the patient and the nature of the clinician's practice (Goldstein *et al*, 2001). As it is not clear which approach for evaluation of the endometrium is more effective, we attempted in this study to find a way of discriminating patients at low and high risk of endometrial cancer. This individualised risk prediction will allow clinicians to make more efficient use of the available diagnostic resources and simultaneously minimise false-negative results from various investigations.

Currently, information gained from the clinical history is not taken into account when performing risk assessment for postmenopausal women with vaginal bleeding. The optimal assessment of women with postmenopausal bleeding would be to stratify the population of women into high-risk and low-risk groups on the basis of history and ultrasound scan results. The low-risk group would undergo endometrial biopsy and the higher risk would undergo immediate visualisation and biopsy of the endometrium for definitive tissue diagnosis.

We propose an algorithm (Norwich DEFAB) for predicting the risk of endometrial carcinoma on the basis of the odds of cancer from multiple logistic regression analysis for individual women presenting with postmenopausal vaginal bleeding. Norwich DEFAB provides a quantitative assessment of the risk of malignancy incorporating patient characteristics of diabetes, ultrasound scan assessment of endometrial thickness, frequency of bleeding, age, and BMI.

We propose that introduction of the Norwich DEFAB probabilistic model in clinical practice can improve the accuracy and efficiency of diagnostic work-up. For women at high risk of malignancy further diagnostic evaluation is indicated even if the initial tests were negative. Depending on prior evaluation, a combination of repeat endometrial biopsy or hysteroscopy should be pursued.

**Chart 1:**
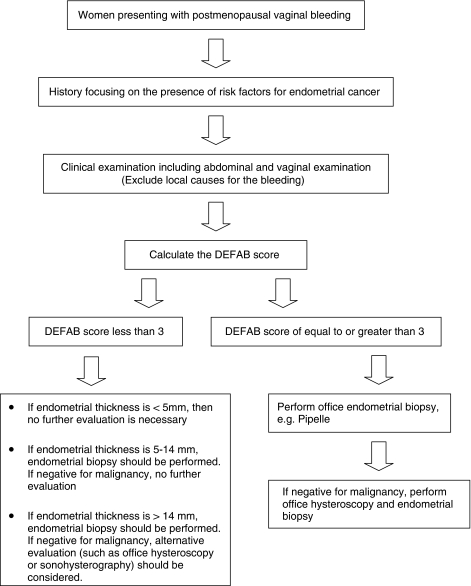
The proposed algorithm for management of women with postmenopausal vaginal bleeding.

For example, a 70-year-old woman with a BMI of 35, who presents with a 2-week episode of vaginal bleeding, would have a Norwich DEFAB score of 6 (age=1, BMI=1, recurrent bleeding=4) if no other risk factors are present. According to current practice, if endometrial thickness measures less than 5 mm on transvaginal ultrasound scan, no further testing would be offered to the patient; only if the patient has an ultrasound scan showing endometrial thickness greater than 5 mm, would an endometrial Pipelle biopsy be performed. However, as this patient is at increased risk of having endometrial malignancy according to the DEFAB score, we suggest that further testing, including endometrial biopsy, should be offered regardless of endometrial thickness measurement. If the biopsy does not show any abnormality, we suggest hysteroscopic evaluation of the endometrium (Chart 1).

We recommend that a Norwich DEFAB cut-off score equal to or greater than 3 should be used to consider further investigations. At this cut-off point, high sensitivity (81.9%) is achieved. Although specificity appears to be low (50.1%), this is not clinically important when considering that the primary objective is not to miss cases of malignancy. A trade-off between sensitivity and specificity is observed as the Norwich DEFAB score increases. For a Norwich DEFAB score of 5 sensitivity decreases to 67.8% and specificity increases to 74.1%.

When developing the predictive model in our study, we analysed type-I and type-II endometrial cancer cases in the same group. Although there are publications showing that women with type-II endometrial cancer have different clinical characteristics when compared with women with type-I endometrial cancer, recent evidence suggests that there is no difference in the age of diagnosis of both types of the disease. Also, obesity increases the risk of both type-I and type-II endometrial cancer (Bjorge *et al*, 2007). In addition, no difference was observed in the results of the predictive model when investigated in women with type-I cancer separately. This was not surprising as we had a small number of women with type-II cancer (21 cases). Further we believe that the model should include all endometrial cancers, as it is not possible to distinguish between the two types at initial presentation of the patient, when applying the algorithm.

One of the limitations of this study is the fact that cases where endometrial thickness measurement was less that 5 mm were attributed to genital tract atrophy and no further investigation was performed. This was a pragmatic study based on the current practice in our unit where transvaginal ultrasonography is used as the initial tool to select patients who require further investigation. This practice is based on the recommendations and evidence mentioned above (ACOG Committee Opinion No 440, 2009). To evaluate the applicability of our findings in other populations, external validation of the predictive model at different cut-off points is required. External validation is also required before introduction of this model in clinical practice.

In conclusion, we have shown that incorporation of clinical information with an initial investigation tool into a risk prediction model allows assessment of the probability of the disease, which may be used to refine subsequent investigations and treatment strategies. This not only has benefit in the process of disease detection but also may result in improved efficiency of care.

It is not yet certain whether application of the Norwich DEFAB in clinical practice will have an effect on the prognosis for endometrial cancer. This should be a topic for further research.

## Figures and Tables

**Table 1:** Basic characteristics of the population. Univariate comparison

**Factors**	**Cancer, *n*=149 (5%)**	**No cancer, *n*=2898 (95%)**	***P*-value**
Age (years)	64 (59–72)	59 (54–67)	<0.0001^a^
BMI (kg m^−2^)	31 (27–36)	28 (25–32)	<0.0001^a^
			
*Diabetes*			
Yes	25 (17%, 11–24%)	158 (5%, 5–6%)	<0.0001^b^
No	124 (83%, 76–89%)	2740 (95%, 94–95%)	
			
*Hypertension*			
Yes	56 (38%, 30–46%)	741 (26%, 24–27%)	0.001^b^
No	93 (62%, 54–70%)	2157 (74%, 73–76%)	
HRT duration (years)	9 (4–20)	5 (2–10)	0.243^a^
			
*Breast cancer*			
Yes	16 (11%, 6–17%)	178 (6%, 5–7%)	0.025^b^
No	133 (89%, 83–94%)	2720 (94%, 93–95%)	
Tamoxifen use (years)	4.5 (2–8)	3 (2–5)	0.091^a^
			
Amount of bleeding*			
Spotting	39 (27%, 20–35%)	611 (21%, 20–23%)	
Light	80 (55%, 46–63%)	1620 (57%, 55–59%)	0.289^b^
Heavy	27 (18%, 13–26%)	614 (22%, 20–23%)	
			
Frequency of bleeding*			
Single	36 (24%, 18–32%)	1541 (53%, 52–55%)	<0.0001^b^
Recurrent	112 (76%, 68–82%)	1345 (47%, 45–48%)	
Endometrial thickness (mm)	14.9 (11.0–21.0)	4.6 (3.0–7.8)	<0.0001^a^

Abbreviations: BMI=body mass index; HRT=hormone replacement therapy.

Values are median (inter-quartile range), number (percent, 95% CI of percent).

a Two-sample Wilcoxon rank sum test (Mann–Whitney test).

b *χ*^2^-Test.

^*^Percentages worked on less numbers from the overall due to missing values.

**Table 2:** Adjusted predictors of cancer (odds ratio) from the best model that fits the data well

**Predictors of cancer**	**Odds ratio (95% confidence interval)**	***P*-value**
Age (years)	1.04 (1.02–1.06)	<0.0001
BMI (kg m^−2^)	1.03 (1.00–1.06)	0.038
Endometrial thickness (mm)	1.15 (1.13–1.18)	<0.0001
		
*Frequency of bleeding*		
Single episode	1	
Recurrent episode	3.93 (2.48–6.23)	<0.0001
		
*Diabetes*		
Yes	1.92 (1.07–3.45)	0.030
No	1	

Abbreviation: BMI=body mass index.

**Table 3:** Overall sensitivity, specificity, and correct classification for each DEFAB cut-off point

**Cut-point**	**Sensitivity**	**Specificity**	**Correctly classified**	**LR (+)**	**LR (−)**	**d-OR**
(>=0)	100.00%	0.00%	4.88%	1.000		—
(>=1)	95.95%	25.57%	29.00%	1.289	0.159	8.11
(>=2)	88.51%	44.70%	46.84%	1.601	0.257	6.23
(>=3)	81.76%	50.28%	51.81%	1.644	0.363	4.53
(>=4)	79.05%	52.56%	53.86%	1.667	0.399	4.18
(>=5)	67.57%	74.43%	74.09%	2.642	0.436	6.06
(>=6)	43.24%	92.38%	89.98%	5.673	0.614	9.24
(>=7)	16.22%	96.92%	92.98%	5.258	0.865	6.08
(>=8)	8.78%	99.27%	94.86%	12.071	0.919	13.13
(>=9)	3.38%	99.90%	95.19%	32.510	0.967	33.62
(>9)	0%	100.0%	95.12%		1.000	—

ROC Area=0.769, 95% CI (0.730–809).

LR (+)=Likelihood ratio (+ve)=Pr (+ve∣+ve)/Pr (+ve∣−ve).

LR (−)=Likelihood ratio (−ve)=Pr (−ve∣+ve)/Pr (−ve∣−ve).

d-OR=diagnostic odds ratio=LR (+)/LR (−).

**Table 4:** Sensitivity, specificity, PPV, and NPV for DEFAB cut-offs of ⩾3 and ⩾5

	**DEFAB score ⩾3, estimate (95% CI)**	**DEFAB score ⩾5, estimate (95% CI)**
Sensitivity	81.9% (74.7–87.7%)	67.8% (59.6–75.2%)
Specificity	50.1% (48.2–51.9%)	74.1 (72.5–75.7%)
ROC area	0.660 (0.627–0.692)	0.710 (0.671–0.748)
PPV	7.78% (6.50–9.21%)	11.9% (9.77–14.2%)
NPV	98.2% (97.4–98.8%)	97.8% (97.1–98.4%)

Abbreviations: CI=confidence interval; NPV=negative predictive value; PPV=positive predictive value; ROC=receiver operating characteristic curve.
